# Does the therapist matter? Therapist characteristics and their relation to outcome in trauma-focused cognitive behavioral therapy for children and adolescents

**DOI:** 10.1080/20008198.2020.1776048

**Published:** 2020-07-23

**Authors:** Elisa Pfeiffer, Silje Mørup Ormhaug, Dunja Tutus, Tonje Holt, Rita Rosner, Tore Wentzel Larsen, Tine K. Jensen

**Affiliations:** a Clinic for Child and Adolescent Psychiatry/Psychotherapy, University Hospital Ulm, University Ulm, Ulm, Germany; b Norwegian Center for Violence and Traumatic Stress Studies (NKVTS), Oslo, Norway; c Division of Mental & Physical Health, Norwegian Institute of Public Health, Oslo, Norway; d Department of Psychology, Catholic University of Eichstätt-Ingolstadt, Eichstätt, Germany; e Centre for Child and Adolescent Mental Health, Oslo, Norway; f Department of Psychology, University Oslo, Oslo, Norway

**Keywords:** therapist, Trauma, PTSD, children and adolescents, TF-CBT, gender match, terapeuta, Trauma, TEPT, niños y adolescentes; TF-CBT, coincidencia de género, 治疗师, 创伤, PTSD, 儿童和青少年, TF-CBT, 性别匹配

## Abstract

**Background:**

There is a broad evidence-base for the effectiveness of Trauma-Focused Cognitive-Behavioral Therapy (TF-CBT) in treating children and adolescents with posttraumatic stress disorder (PTSD). The effect of therapist characteristics on patient symptoms has been neglected in psychotraumatology research and necessitates further investigation.

**Objective:**

This study aims to investigate the impact of therapist characteristics (gender, clinical experience and theoretical background) on posttraumatic stress symptoms (PTSS) in a heterogeneous and international sample of traumatized children and adolescents.

**Method:**

Data from two RCTs on the effectiveness of TF-CBT in Norway and Germany were included, comprising *N* = 52 therapists (78.8% female) and *N* = 153 patients (72.3% female). All therapists underwent thorough training and continuous supervision in TF-CBT. The Clinician-Administered PTSD Scale for Children and Adolescents (CAPS-CA) assessed pre- and post-treatment served as the outcome variable in a linear mixed-effects model with therapists’ theoretical background, prior clinical experience and gender as independent variables, adjusted for patient gender, measurement time and country. The possibility of an interaction between therapist and patient gender was investigated subsequently.

**Results:**

None of the therapist characteristics were significantly related to PTSS. There was no indication of an interaction between patient and therapist gender (*p* =.878).

**Conclusion:**

The lack of evidence for a relationship of therapists’ theoretical orientation and clinical experience with outcome suggests that a wider dissemination of TF-CBT may be warranted. More studies with larger therapist and patient sample sizes and including only community practice are needed.

## Abstract (es)

**Antecedentes**: Hay una amplia base de evidencia de la efectividad de la Terapia Cognitivo Conductual con foco en el trauma (TF-CBT) en el tratamiento de niños y adolescentes con Trastorno de Estrés Postraumático (TEPT). El efecto de las características del terapeuta sobre los síntomas del paciente ha sido dejado de lado en la investigación en psicotraumatología y requiere mayor investigación.

**Objetivo**: Este estudio apunta a investigar el impacto de las características del terapeuta (género, experiencia clínica y conocimiento teórico) sobre los síntomas de estrés postraumático (PTSS) en una muestra heterogénea e internacional de niños y adolescentes traumatizados.

**Método**: Se incluyeron datos de dos ERC (RCTs) acerca de la efectividad de la TF-CBT en Noruega y Alemania, que constan de *N*=52 terapeutas (78.8% mujeres) y *N*=153 pacientes (72.3% mujeres). Todos los terapeutas se sometieron a entrenamiento y supervisión contínua en TF-CBT. La Escala de TEPT administrada por clínicos para niños y adolescentes (CAPS-CA) aplicada pre y post tratamiento sirvió como variable de resultado en un modelo lineal de efectos mixtos con el conocimiento teórico del terapeuta, su experiencia clínica previa y género como variables independientes, ajustada por género del paciente, tiempo de medición y país. La posibilidad de una interacción entre el género del terapeuta y del paciente se investigó posteriormente.

**Resultados**: Ninguna de las características del terapeuta se relacionaron significativamente a PTSS. No hubo indicios de una interacción entre el género del paciente y del terapeuta (*p*=.878).

**Conclusión**: La falta de evidencia para una relación entre la orientación teórica y experiencia clínica del terapeuta y el resultado sugiere que se puede requerir una más amplia diseminación de TF-CBT. Se requiere mós estudios, con muestras de mayor tamaño de pacientes y terapeutas y que incluya sólo práctica comunitaria.

## Abstract (zh)

**背景：**聚焦创伤认知行为疗法（TF-CBT）在治疗患有创伤后应激障碍（PTSD）的儿童和青少年中的有效性具有广泛的证据基础。心理创伤学研究忽略了治疗师特征对患者症状的影响，需要进一步研究。

**目的：**本研究旨在考查一个具有异质性和国际性的创伤儿童和青少年样本中，治疗师特征（性别、临床经验和理论背景）对创伤后应激症状（PTSS）的影响。

**方法：**纳入挪威和德国TF-CBT有效性的两个RCT数据，其中包括52位治疗师（78.8％女性）和153位患者（72.3％女性）。所有治疗师都接受了TF-CBT的全面培训和持续监督。在线型混合效应模型中，治疗前后临床用儿童和青少年PTSD量表（CAPS-CA）的评估作为结果变量，治疗师的理论背景、既往临床经验和性别为自变量，以患者性别、测量时间和国家作为调节。随后研究了治疗师与患者性别之间交互作用的可能性。

**结果：**没有任何治疗师特征与PTSS显著相关。没有迹象表明患者和治疗师性别之间存在交互作用（*p* =.878）。

**结论：**缺乏证据表明治疗师的理论取向和临床经验与结果之间存在关联，这表明可能需要更广泛地传播TF-CBT。需要更多治疗师和患者样本量较大且仅包括社区实践的研究。

## 1. Introduction

By the time they reach adulthood, up to 56% of children and adolescents living in Europe have experienced at least one potentially traumatizing event (Landolt, Schnyder, Maier, Schoenbucher, & Mohler-Kuo, 2013; Lewis et al., 2019; Perkonigg, Kessler, Storz, & Wittchen, 2000). In the aftermath, about 16% develop posttraumatic stress disorder (PTSD; Alisic et al., 2014), depending on trauma type, severity and gender (Trickey, Siddaway, Meiser-Stedman, Serpell, & Field, 2012). PTSD in childhood can significantly hamper an individual’s functional level and quality of life in both the short and long term. Trauma treatment is, therefore, crucial. International treatment guidelines (International Society for Traumatic Stress Studies [ISTSS], 2019; National Institute for Clinical Excellence [NICE], 2018) recommend ‘Trauma-Focused Cognitive-Behavioral Therapy’ (TF-CBT; Cohen, Mannarino, & Deblinger, 2016) as the first-line treatment for children and adolescents suffering from PTSD as it is the most researched and consistently efficient treatment for PTSD in children and adolescents. In spite of broad empirical support for the feasibility and effectiveness of TF-CBT in different contexts (e.g. Morina, Koerssen, & Pollet, 2016; Murray et al., 2015), a significant proportion of youth do not respond to the treatment sufficiently (Knutsen, Sachser, Holt, Goldbeck, & Jensen, 2019). There may be numerous, patient related reasons for different responses to treatment such as neurological and biological factors (Bryant et al., 2008), patients’ treatment expectations (Mohr, 1995), comorbid disorders (Vittengl, Clark, Smits, Thase, & Jarrett, 2019), or demographic characteristics such as the patients’ gender (Knutsen et al., 2019).

To better understand the variation in therapeutic outcomes, several researchers have turned their attention to therapist factors (e.g. Heinonen & Nissen-Lie, 2019). As Johns, Barkham, Kellett, and Saxon (2019) argue in their recent review, the investigation of therapist effects is highly relevant, as more and less effective therapists could be identified. This, in turn potentially enables better matching of therapist and patient, and more effective treatment. Knowledge about therapist effects on outcome can further stipulate more research aiming to reduce variability between therapists in an effort to improve overall service performance.

The majority of studies, which mostly investigated adult patients, report moderate therapist effects of approximately 5% (Johns et al., 2019), but the overall range of therapist effects is relatively large and varies from 0.2% to 48.7% (Crits-Christoph et al., 1991; Crits-Christoph & Mintz, 1991; Johns et al., 2019; Lutz, Leon, Martinovich, Lyons, & Stiles, 2007). Frequently researched therapist variables are the therapists’ theoretical background/orientation (Anderson, Ogles, Patterson, Lambert, & Vermeersch, 2009; Berglar et al., 2016; Okiishi et al., 2006), professional interpersonal skills or self-rated professional characteristics (Heinonen & Nissen-Lie, 2019), therapists’ alliance-building behaviours (Jungbluth & Shirk, 2009; Ovenstad, Ormhaug, Shirk, & Jensen, 2020), professional experience (e.g. years of experience doing therapy; Huppert et al., 2001; Turner, Nicholson, & Sanders, 2011), age (Anderson et al., 2009; Schauenburg, Dinger, & Strack, 2005) and gender (Shiner, Westgate, Harik, Watts, & Schnurr, 2017; Zorzella, Muller, & Cribbie, 2015). However, these studies have investigated very heterogeneous samples and the results regarding the impact of the therapist variables on outcome are highly contradictory. For example, some of the studies that have looked at the relevance of therapists’ professional clinical experience have not found a significant effect on outcome (Andersson, Carlbring, Furmark, & the SOFIE Research Group, 2012; Dinger, Strack, Leichsenring, Wilmers, & Schauenburg, 2008; Okiishi et al., 2006; Turner et al., 2011), whereas other studies with adults have found that training experience in the mental health field was associated with better treatment results (Abrahamson, Arling, & Gillette, 2012; Berglar et al., 2016; Huppert et al., 2001). This implies that therapist age may be related to better outcome. Younger therapists, however, were reported to use manual-based treatments more often than more experienced therapists (Becker, Smith, & Jensen-Doss, 2013) which may in turn lead to better treatment results. In light of these mixed findings, the relationship between experience and outcome warrants further research.

Few therapist studies have focused on therapists delivering trauma-focused treatments in general and on child treatment in particular (Baldwin & Imel, 2013; Johns et al., 2019). Ruzek et al. (2016) investigated several treatment provider characteristics in 943 mental health professionals treating veterans with PTSD in the USA who received training in prolonged exposure therapy (PE; Foa & Rothbaum, 1998). They found that providers who identified with a cognitive behavioural therapy (CBT) background were more likely to complete consultation with their supervisors (*p* = .001). Another study by Shiner et al. (2017) looked at the influence of therapist characteristics and gender matching on retention rates in a national cohort of US veterans with PTSD. They found that younger therapist age was associated with higher retention rates among patients. Regarding the gender match between therapist and patient, it was not a significant predictor for retention when other patient and therapist factors were adjusted for.

Since most of the studies on the role of therapists are conducted with adult samples, little is known about therapist effects in psychotherapy with children and adolescents (Fjermestad, McLeod, Tully, & Liber, 2016). So far, only preliminary results on single factors such as therapists’ relationship skills (Alexander, Barton, Schiavo, & Parsons, 1976), therapists’ alliance building behaviours (Jungbluth & Shirk, 2009; Ovenstad et al., 2020) or ethnicity and language of the therapist are available (Halliday-Boykins, Schoenwald, & Letourneau, 2005; Yeh, Eastman, & Cheung, 1994). A study by Wintersteen, Mensinger, and Diamond (2005) found that ‘same gender’ matched dyads of therapists and patients in the treatment of 600 adolescent substance abusers reported higher alliance ratings and that the patients were more likely to complete treatment compared with ‘different-gender’ dyads. Most studies, however, looked at retention rates or alliance, not symptoms, as outcome (De Haan, Boon, de Jong, Hoeve, & Vermeiren, 2013). To our knowledge, there are no studies that have examined the impact of therapist characteristics in trauma-focused treatments for children and adolescents.

In TF-CBT, the only therapist-related factor that has been researched to any extent is the working alliance between patient, therapist and caregiver (Kirsch, Keller, Tutus, & Goldbeck, 2018). The alliance between patient and therapist has been proven to have a significant impact on therapy outcome (Ormhaug, Jensen, Wentzel-Larsen, & Shirk, 2014) and female therapists seem to have higher rates of alliance with their patients (Zorzella et al., 2015).

In sum, taking a look at the characteristics of therapists delivering the trauma treatment, with the most evidence for traumatized children and adolescents, seems inevitable in order to not only gain a better understanding of different treatment responses, but also to improve trauma care overall.

### 1. 1. Study aim and hypotheses

This secondary analysis of two RCT studies in Norway (Jensen et al., 2014) and Germany (Goldbeck, Muche, Sachser, Tutus, & Rosner, 2016) aims to evaluate the effect of different therapist factors in a European sample of children and adolescents receiving TF-CBT. In particular, the relationships of therapists’ gender, theoretical background and years of clinical experience with PTSS will be investigated, adjusted for patient gender, measurement time and country. Due to the scarce and contradictory literature, analysis will be conducted without directed hypotheses. The possibility of an interaction between therapist and patient gender will be explored subsequently.

## 2. Methods

### 2. 1. Procedure

The Norwegian study was approved by the Regional Committee for Medical and Health Research Ethics (ClinicalTrials.gov Identifier: NCT00635752). The German study was approved by the IRB at the University of Ulm (12/08 and 192/13; ClinicalTrials.gov Identifier: NCT01516827). The recruitment phases were from April 2008 to February 2011 in Norway, and from February 2012 to January 2015 in Germany.

### 2. 2. Patient and therapist sample

Only participants in the TF-CBT conditions were included in this secondary analysis of both trials. Hence, the Norwegian subsample consisted of *n* = 79 (*M*_age_ = 15.03 years; *SD_age_* = 2.20 years; range 9–18 years, 74.7% girls; 75.9% interpersonal trauma) children and adolescents and the German subsample consisted of *n* = 74 (*M*_age_ = 12.73 years, *SD_age_* = 2.91 years; range 7–18 years; 68.9% girls; 79.7% interpersonal trauma) children and adolescents.

TF-CBT was delivered by a total of 52 study therapists, *n* = 26 in Germany (Goldbeck et al., 2016) and *n* = 26 in Norway (Jensen et al., 2014) (see Table 1). The therapists were mostly female (78.8%), *n* = 26 (50%) were born in Norway, *n* = 19 (36.5%) in Germany, *n* = 3 (5.8%) in other European countries and for *n* = 4 (7.7%) the country of origin is unknown. They had on average 9.34 years of clinical experience (*SD* = 7.14; range 1–31 years). Regarding their theoretical background, *n* = 35 (67.3%) had a CBT background, *n* = 8 (15.4%) a psychodynamic background and *n* = 6 (11.5%) a systemic/family background. All the therapists read the TF-CBT manual, participated in a certified web-based training (tfcbt.web), and attended a two-day training course run by a trainer certified by the developers of TF-CBT. In addition, the therapists were continuously supervised at their clinics and were invited to participate in bi-monthly case-based conference calls with certified TF-CBT trainers.

**Table 1.** Socio-demographic data of patients and therapists.

		Patients *N* = 153	Therapists *N* = 52	
Age (years), *M* (*SD*)		13.9 (2.8)	n/a	
Gender, *n* (%)				
	Male	43 (28.1)	11 (21.2)	
	Female	110 (71.9)	41 (78.8)	
Theoretical orientation, *n* (%)				
	CBT		35 (67.3)	
	Psychodynamic		8 (15.4)	
	Systemic/Family therapy		6 (11.5)	
	Unknown		3 (5.8)	
Clinical experience (years), *M(SD)*			9.3 (7.1)	

n/a means data not available.

### 2. 3. Intervention

TF-CBT is a short-term, component-based intervention consisting of 12 to 15 weekly 90-minute sessions (Cohen et al., 2016). Each component is offered to the child and the caregiver (mostly parent) in both parallel and conjoint sessions. The nine components (psychoeducation and parent training, relaxation, affect modulation, learning cognitive coping skills, trauma narrative, cognitive processing of the trauma, in vivo mastery of trauma reminders, enhancing safety, and future development) constitute three treatment phases: 1) stabilization and skill building, 2) exposure and cognitive processing of the trauma, and 3) fostering safety and future development. Adherence to the manual was high in Germany (96%) and Norway (94%). The adherence to the model was ascertained by an independent review of the complete first study case and at least 25% randomly selected video-taped sessions in the German study and 100% of audio-taped sessions in the Norwegian study.

### 2. 4. Measurements

All study therapists filled out demographic questionnaires on their age, gender, clinical experience, and theoretical background.

The frequency and intensity of posttraumatic stress symptoms (PTSS) was assessed using the *Clinician-Administered PTSD Scale for Children and Adolescents* (CAPS-CA; Nader et al., 1996) in both studies pre- and post-treatment. This served as the outcome variable. The CAPS-CA is a clinical interview that measures trauma exposure along with the frequency and intensity of the 17 DSM-IV-defined symptoms of PTSD (American Psychiatric Association, 2000). Items are rated on 5-point frequency scales (0 = ‘none of the time’ to 4 = ‘most of the time’) and 5-point intensity scales (0 = ‘not a problem’ to 4 = ‘a big problem’) for the previous month (possible range: 0–68). The internal consistency of the symptom score was α = .90 in the Norwegian study sample (Jensen et al., 2014) and α = .79 in the German sample (Goldbeck et al., 2016).

### 2. 5. Statistical analysis

The analyses included all participants within the TF-CBT condition in Norway and Germany. The data were analysed using mixed-effects models with therapist as the first clustering level, and measurement time points for each patient (CAPS-CA pre- and posttreatment) as the second clustering level. Random structures were investigated for stability and simplified when necessary as recommended by Pinheiro and Bates (2000). Specifically, models for which it was impossible to compute confidence intervals for the random effects or where the lower bound was extremely low or the upper bound extremely high, were considered as unstable.

The factors theoretical background, therapists’ gender and years of clinical experience were included in the model, with adjustment for patient gender, country in which the study was conducted (Germany vs. Norway) and time of measurement. For this analysis, *n* = 7 participants had to be excluded due to missing data on therapist’s clinical experience (*n* = 4), therapists’ theoretical background (*n* = 1), or both (*n* = 2). The model was re-run with the inclusion of an interaction between therapist and patient gender, in order to check for possible differences between gender-based therapist and patient dyads. The significance level was set at *p* = .05 (2-tailed) for all analyses. All analyses were done with R version 3.6.1 (The R Foundation for Statistical Computation, Vienna, Austria) and the R package nlme (Pinheiro & Bates, 2000) for mixed-effects models.

## 3. Results

The mixed-effects model with patients nested within therapists was unstable. This is probably due to the small number of patients treated for some therapists (see Table 2). We, therefore, simplified the random structure by deleting the therapist clustering level, resulting in a linear mixed-effects model with only one clustering level: The two time points for each patient (CAPS-CA T1 and CAPS-CA T2). The results (see Table 3) show that neither patient gender, nor theoretical background, nor years of clinical experience were significantly related to CAPS-CA. The gender of the therapist, however, was close to significantly related to outcome, as patients treated by a male therapist reported lower CAPS-CA scores than patients treated by a female therapist. There was no indication of an interaction between therapist and patient gender (*F*(1,139) = .02; *p* = .878).

**Table 2.** Number of cases per therapist.

Cases (*n*)	Number of therapists (*n*)
1	16
2	12
3	9
4	6
5	4
6	3
11	1
13	1

**Table 3.** Results of the linear mixed effects model of therapist variables on treatment outcome (posttraumatic stress symptoms).

Variable	Difference			95% CI	*p*
Theoretical orientation (CBT)	−4.52			−11.48 to 2.43	.200
Years of experience	.03			−.43 to .48	.906
Therapist gender (female)	6.99			−.53 to 14.51	.068
Patient gender (girl)	6.35			.07 to 12.63	.047
Country (Germany)	−.37			−6.79 to 6.05	.909
Time (T2)	−30.61			−34.70 to −26.52	<.001

The variables patient gender, country in which the study was conducted (Germany vs. Norway) and time of measurement (Time (T2)) were only added as control variables.

## 4. Discussion

This is the first study to evaluate the effect of therapist characteristics on patient outcome in traumatized children and adolescents receiving evidence-based trauma-focused treatment. We did not find any statistically significant effects of theoretical background and prior clinical experience of therapists on PTSS, which is only partially in line with prior research (Abrahamson et al., 2012; Berglar et al., 2016; Huppert et al., 2001). There are two possible explanations for this finding: The first explanation might be a power problem. Although the sample of therapists exceeded the recommended minimum of 50 (Johns et al., 2019), the number of patients treated by these therapists might have been just too small and unbalanced. Schiefele et al. (2017) suggest having at least four patients per therapist, which was not achieved in this sample. The potential power problem of this study seems to be an overall issue in therapist effect research, especially when investigating a specific treatment or study population. In Baldwin and Imel’s review (2013), 43 out of 46 studies can be classified as having serious sample size problems (*Md*_therapist number_ = 9 [7.6 patients per therapist]). Moreover, Johns et al. (2019) found in their review that, in the sample comprising RCTs and practice-based studies, the median number of therapists per study was 57.5 and in 12 (60%) of the studies the number of patients per therapist exceeded 30. Future studies should not only investigate the role of therapists in evidence-based treatments (EBTs) but also do so with a more balanced therapist-patient allocation and, potentially, with larger (therapist and patient) samples. The second explanation for the non-significant differences between therapist characteristics might be the naturally high standards of the RCT design in which the investigators try to ensure that only the treatments differ. Therapists in both RCTs were self-selected and might, therefore, have been particularly interested and engaged, resulting in an overall ceiling effect. Independent of theoretical background and prior clinical experience, all therapists in this study underwent the same extensive training and continuous clinical supervision by TF-CBT trainers. Moreover, fidelity ratings were high which means that differences between therapists might have been compensated as all therapists adhered to the manual. This is in line with a meta-analysis of 27 separate treatment groups. The study found that the use of a treatment manual was associated with smaller differences between therapists compared to treatments not using a manual (Crits-Christoph et al., 1991).

The results show that only therapist gender was close to significantly related to symptoms. Although this result was not statistically significant, there was a trend that patients treated by male therapists reported less PTSS compared to patients treated by female therapists. This is contrary to prior research conducted with adult samples diagnosed with panic disorder (Huppert et al., 2001) or major depressive disorder (Zlotnick, Elkin, & Shea, 1998). We can only speculate why male therapists may seem to outperform female therapists in our study. A further investigation showed that on average, the male therapists’ patients had lower pre-treatment PTSS scores compared to the patients of female therapists (*p = *.027). However, this was to some extent taken into account since the PTSS pre-treatment scores were part of the dependent variable in each model. Another explanation could be the sample distribution in this study, as two of the male therapists treated more patients than the rest of the male therapist (i.e., 11 and 5 patients each, whilst the rest of the male therapists (*n* = 9) treated 1–3 youth). Therefore, the difference could be related to these two therapists’ specific therapist skills rather than their gender. However, an investigation of the results show that there was no statistically significant difference in the results of the two therapists compared to the rest of the male therapists (see supplementary materials A). A third possibility could be that therapist gender gives potential for a corrective emotional experience. In the present study sample, many patients had experienced interpersonal violence (77.78%). Even though we do not have information about the gender of the perpetrator in the present study, it may be that, since studies have found that the majority of perpetrators are male (Finkelhor, Ormrod, & Chaffin, 2009), having a sensitive and caring male therapist might not only counteract avoidance but also reduce dysfunctional appraisals of men as dangerous. These dysfunctional appraisals play a key role in PTSS development and maintenance (Ehlers & Clark, 2000; Meiser-Stedman, Dalgleish, Glucksman, Yule, & Smith, 2009), and are proven to be a change mechanism during treatment (Jensen, Holt, Mørup Ormhaug, Fjermestad, & Wentzel-Larsen, 2018; Pfeiffer, Sachser, de Haan, Tutus, & Goldbeck, 2017). Future research needs to replicate these findings and investigate the effect of therapist gender with larger samples in order to draw valid clinical implications.

Although there is no clear evidence of a statistically significant therapist gender difference in this study, the trend in that direction warrants further studies that could determine if there is evidence of a difference with a larger sample of (male) therapists and, if so, whether the possible explanations described above might be responsible for that.

### 4. 1. Strengths and limitations

This study is the first study to look at therapist characteristics in the field of EBTs for traumatized children and adolescents by analysing two well-conducted RCT studies in two different European countries. In fact, only three studies have evaluated therapist effects in RCTs so far (Erickson, Tonigan, & Winhusen, 2012; Goldsmith, Dunn, Bentall, Lewis, & Wearden, 2015; Moyers, Houck, Rice, Longabaugh, & Miller, 2016). They investigated: substance abuse, chronic fatigue syndrome and alcohol-related difficulties. The present study took into account very different theoretical backgrounds and rather non-experienced next to very experienced therapists. Consequently, this therapist sample is quite representative for clinical practice. Also, this study was conducted in both specialized and non-specialized clinics and included a heterogeneous sample of traumatized children and adolescents. They represent typical trauma cases in the German and Norwegian mental health care systems. Most importantly, the study investigated therapists delivering TF-CBT, which is now being rolled out in many countries around the world.

Despite these strengths the results need to be viewed in the context of some limitations: (1) The authors were not able to perform hierarchical analysis with patients nested in therapists due to the patient-therapist allocation. (2) The study included a rather small patient/therapist sample. (3) There was an uneven allocation of patients to therapists. However, we could not find significant differences between therapists who treated one or two cases and therapists who treated more than two cases at pre- and post-treatment PTSS scores (see supplementary materials B). (4) Although demographic information was comparable between therapist gender groups (see supplementary material C), the CAPS-CA baseline scores of patients treated by male therapists were significantly lower (*p* = .027) compared with patients treated by female therapists (see supplementary material D). This was taken into consideration as baseline scores were part of the dependent variable. (5) The small sample size particularly of male therapists may have limited the statistical power to test for a gender difference. (6) The therapists in this study were relatively inexperienced (*M* = 9.34 years; *SD* = 7.14 years). Hence, the results of this study might mainly apply to therapists with 15 or fewer years of clinical experience, although a small number of the participating therapists (*n* = 7; 13%) did have 16–31 years of clinical experience. (7) The findings are generalizable for patients entering routine clinical care in which interpersonal trauma is highly frequent, but not for samples who experience mostly accidental trauma. (8) Due to high levels of standardization and adherence to the manual in this study, the implementation of TF-CBT might not sufficiently represent the work of therapists in regular practice outside of the study context. In both RCT studies, all therapists received regular supervision and therefore factors such as theoretical orientation and years of clinical experience may play a more salient role in the deliverance of TF-CBT with low to no levels of supervision.

### 4. 2. Clinical implications

The study results are encouraging for clinical practice as they suggest that, as long as the treatment provider is trained, supervised and has a thorough understanding of the manual, he/she might be capable of delivering trauma-focused treatment with good outcome results. This study might, therefore, counteract myths regarding PTSD treatment in general and exposure in particular (Feeny, Hembree, & Zoellner, 2003) and encourage practitioners to deliver trauma-focused EBTs which normally include exposure. At the present time, the utilization of exposure therapy in PTSD is still not widely accepted or used by practitioners (Levita, Duhne, Girling, & Waller, 2016). Also for researchers, the study results are important, as they might motivate researchers to further disseminate and train trauma-focused EBTs to all therapists around the world.

### 4. 3. Conclusion

The latest reviews of therapist effects agree that therapist characteristics are largely neglected in therapy process research (Fjermestad et al., 2016; Karver, Handelsman, Fields, & Bickman, 2005, 2006). There is very little and highly contradictory findings on the role of the therapist characteristics in the treatment of child and adolescent samples receiving evidence-based trauma-focused treatments. The high standardization process within the study design might, in fact, be responsible for the non-significant differences between therapists. More studies with sufficient therapist and patient sample sizes in community practice are needed to investigate the effect of therapist characteristics on outcome. The results of this study can be perceived as a stepping stone in understanding the therapeutic change in trauma-focused youth therapy. However, it should be seen as a small glimpse of larger theoretical models which include various components hypothesized to relate to patient outcome. Hopefully this study will be beneficial in disseminating EBTs by making a case that the therapist’s prior clinical experience and theoretical background might not be crucial for delivering exposure-based manualized treatment.

## Supplementary Material

Supplemental Material
