# Supplementary material for: Does the therapist matter? Therapist characteristics and their relation to outcome in trauma-focused cognitive behavioral therapy for children and adolescents
Source: Eur J Psychotraumatol. 2020 Jul 23;11(1):1776048. doi: 10.1080/20008198.2020.1776048 (PMC7473296; doi:10.1080/20008198.2020.1776048)
Supplement: Supplemental Material [file ZEPT_A_1776048_SM3192.docx]

Supplementary Materials

Table A

*Differences in posttraumatic stress symptoms (PTSS; CAPS-CA) between male therapists who treated five or eleven cases and therapists who treated less patients.*

|  | Number of cases grouped | | | | Group comparisons | | | |
| --- | --- | --- | --- | --- | --- | --- | --- | --- |
|  | therapists with 5 or 11 cases | | therapists with less cases | |  | | | |
|  | n | M (SD) | n | M (SD) |  | Student t-Test | | 95% CI |
| Pre-treatment PTSS | 16 | 56.38 (17.16) | 15 | 48.33 (14.97) |  | | *t* (29) = 1.39;  *p* = .176 | [-3.82, 19.90] |
| Post-treatment PTSS | 16 | 19.75 (19.36) | 9 | 18.89 (18.64) |  | *t* (23) = .11; *p* = .915 | | [-15.61, 17.33] |

Table B

*Differences in posttraumatic stress symptoms pre- and post-treatment (CAPS-CA) between therapists who treated one or two cases and therapists who treated more than two* *cases*

|  | Number of cases grouped | | | | Group comparisons | | |
| --- | --- | --- | --- | --- | --- | --- | --- |
|  | 1 or 2 cases | | more than 2 cases | |  | | |
|  |  | M (SD) |  | M (SD) | Student t-Test | 95% CI |  |
| Pre-treatment PTSS |  | 58.79 (17.72) |  | 59.05 (18.63) | *t* (151) = -.08; *p* = .939 | [-7.07, 6.54] |  |
| Post-treatment PTSS |  | 30.04(27.91) |  | 27.77 (23.42) | *t* (115) = .40; *p* = .688 | [-8.94, 13.49] |  |

Table C

*Therapist Characteristics sorted by gender.*

|  | | |  | Gender | |  |
| --- | --- | --- | --- | --- | --- | --- |
|  | | |  | Male | Female | Group comparisons |
|  | | |  | n | n |  |
| Theoretical background | | |  |  |  | *χ^2^*(1) = .75; *p* = .386 |
| CBT | |  | 9 | 26 |  |  |
| Other | |  | 2 | 12 |  |  |
| Systemic/Family Therapy |  | 1 | 5 |  |  |  |
| Psychodynamic |  | 1 | 7 |  |  |  |
| Years of clinical experience | | |  | | |  |
| M (SD) | |  | 11.14 (9.94) | 8.79 (6.12) | *t*(45) = .74; *p* = .473 |  |
| Minimum | |  | 2.5 | 1 |  |  |
| Maximum | |  | 31 | 28 |  |  |

Table D

*Differences Therapist Gender on pre-treatment PTSS (CAPS-CA T1) and post-treatment PTSS (CAPS-CA T2)*

|  | Therapist gender | | | | Group comparisons | |
| --- | --- | --- | --- | --- | --- | --- |
|  | Male | | Female | |  | |
|  |  | M (SD) |  | M (SD) | Test | 95% CI |
| Pre-treatment PTSS |  | 52.48 (16.38) |  | 60.64 (18.51) | *t* (151) = -2.24;  *p* = .027 | [-15.35, -.96] |
| Post-treatment PTSS |  | 19.44 (18.71) |  | 30.60 (25.11) | *t* (115) = -2.07;  *p* = .041 | [-21.84, -.47] |
